# Glycogenia—The Secretion of Sugar in the Human Economy

**Published:** 1856-01

**Authors:** Bernard Henry


### *Glycogenia—The Secretion of Sugar in the Human Economy.* By Bernard Henry, M. D.

The present would seem to be an opportune moment for the examination of the question of the production of sugar in the system, and its physiological and pathological import.

It is now nearly six years since Mr. Claude Bernard announced to physiologists, that sugar is a normal secretion in man and animals, and maintained, by experimental proof, that this function has its seat in the liver. The disease, diabetes mellitus, in which grape sugar appears in the urine, had been studied and observed with much attention ever since the time of Willis, in 1674, but its pathology was still obscure, and various causes had been assigned to it.

The question, as it now stands, has been placed before the world in the work just published by the distinguished teacher above mentioned:

"Lectures on Experimental Physiology applied to Medicine." Its appearance had been preceded by some controversial writings on the subject it embraces, and it is interesting to examine and ascertain what position its doctrines occupy at present.

It was in an Inaugural Thesis, published in December, 1843, that Mr. Bernard first made known his researches on the production and destruction of sugar in the system. Like many other discoveries in science, the first *facts* on this subject presented themselves during the investigation of other ph enomena. It is in this manner that inductive and experimental physiology has so much and so frequently contributed to the development of medicine.

Chemistry had insisted that all the principles met with in the organism must be traced from substances, capable of corresponding metamorphoses, by the formula, or in the laboratory of the chemist; and that thus every organic compound to be formed in that body, should be a derivative by oxidation or by re-arrangement of atoms, of that which had been formed exteriorly; that the animal fabric was capable only of destructive metamorphosis, and that the saccharine principles occurring in the economy owed their origin only to the vegetable constituents of the food. It was from this, that Prout assigned the presence of sugar in diabetus mellitus to the imperfect transformation and assimilation of the starchy elements of nutrition, considered it a defect of the digestive functions, and called away the attention of pathologists from the kidneys, which had been previously accused in this disease. These organs, which had so frequently been found inflamed, diseased, and even destroyed, worn out by their efforts to eliminate a substance that was in excess in the blood, had been regarded as primarily at fault, and responsible for all the phenomena of the disease; the other organs were not made the objects of proper examination. It is physiology which has taken up the task and done so much for this interesting subject; but it is yet incomplete; the knife of the experimenter, it is true, has laid bare the seat of the production of animal sugar, and has shown us where it always exists in the system in health; it is the scalpel of the pathological anatomist, now, that must continue the work, and point out the leisons in the diseases with which the subject is associated; one which appears to be singularly deficient in records of the post mortem observations, as well as intractable by known remedies; and it is to call attention to these circumstances that our remarks are made.

As regards the internal production of sugar in animals without the intervention of saccharine or amylaceous food, that a substance, previously supposed to be elaborated only in the plant, should be formed in animals, does not, at the present day, appear singularly remarkable, since the production of cellulose, a vegetable tissue in the structure of lower animals, and even in the nervous system of man, is now a well-admitted fact. (Virchow.)

The first proposition announced by Bernard was, that in dogs which had for months been fed solely on meat and then killed, glucose (grape sugar) was found in the blood of the hepatic vein, and in the liver, but could not be detected in the blood of the vessels leading to that organ. Hence, he drew the deduction that the liver is the laboratory for its production, as well as the demonstration of the fact that sugar can originate in the organism, independently of an external source; that is, of sugar-yielding vegitable aliment; that it is a true secretion, and as later experiments have proved, subordinate, as the functions of other glands are, to the nervous influence.

The points then to be inquired and determined were: What proofs have we that the liver is the sole producing organ? what are the materials and conditions for its production? what physiological office does glucose perform in the economy? what are the causes of its accumulation in the blood and appearance in the urine in disease; and from these facts to form a rational plan of treatment of diabetes mellitus.

In 1778, Cowley first isolated the saccharine principle of diabetes; but it was not until 1815 that the demonstration was made by Chevreuil, that this sugar is identical with the sugar of fecula, or grape sugar, belonging to a class which forms a separate groupe from those which are represented by cane sugar.

These facts are all recognizable by the caustic alkalies, by the addition of which and heat, they are converted into peculiar brown acids; those of the outer class are not thus affected. This, however, is not a good test in fluids containing organic substances, as the blood and urine.

Trommer ascertained that glucose has the property of decomposing certain salts of copper in the presence of an alkali. If the liquor be heated, the oxide of copper is precipitated of a characteristic yellow orange, or brick dust color; he ascertained, also, that this does not occur with cane sugar.

A modification of this, consisting of a solution of the double tartrate of copper and potassa, has been produced by Barreswill, and is the one recommended and used by Bernard in his experiments. If this be added to a solution of glucose, it loses its blue color, and on heating the liquid, there is formed the yellowish red precipitate of the oxide of copper. The quantity of the test fluid to be added, should be in proportion to the amount of glucose expected to be present, in order that reduction of the salt of copper be complete, and the characteristic color obtained.

It should be borne in mind, that cane sugars are by acids converted into grape sugars, so that if an acid have found its way into the mixture, this test would reveal also the occurrence of sugars of that class.

Charcoal is the best means of decolorizing any fluid, and freeing it of all substances; as uric acid, dextrine, &c., which might interfere with the reactions of the double salt of copper. In consequence of the objections that have been made to these tests, and which will be considered shortly, it is important and desirable to employ the fermentation test, by the addition of yeast to the suspected liquid, for the production of alcohol and carbonic acid from the glucose present.

The presence of sugar in the liver is now an undeniable fact. It may be made apparent with facility in a decoction of the viscus, by any of the above tests, as has been frequently done by the writer. This glycogenic function would appear to exist in all the animals as well as man, thus far examined. Its proportion in the physiological state does not vary much. The mean is about two per cent. for the entire weight of the liver, in mammalia and birds. It is said to be less in reptiles, and still less in the mollusca. Its existence may easily be determined in these last, by using several livers, making a concentrated decoction, decolorizing by charcoal or sulphate of soda, and employing the tests of copper, fermentation, &c.

The animals must be killed in full health, as in chronic diseases, shortly before death, this function of the liver, like those of the other organs, ceases. It is for this reason that the livers of subjects in the anatomical rooms will not yield glucose. Mr. Bernard has had the opportunity of examining the livers of six persons who had met with violent deaths whilst in full health, executed criminals, &c. In all of them, the liver yielded glucose, which could not be detected in any of the other organs.

The liver, then, is the only organ furnishing this substance, and the question naturally arises, whence doth it obtain it? does it originate in its tissue, or is it carried there in its blood? The inference drawn from the capital experiment of Bernard, viz: that the blood of the vessels entering the liver did not yield glucose, whilst that leaving that viscus yielded it freely, and which would seem to point out, with precision, the starting point of this principle, has not been admitted without modification and opposition.

According to the experiments of Poggiale, a dog fed on a mixed diet, or a diet of bread alone, and then killed, gave evidences of glucose in the blood of the portal vein, in that of the inferior cava, and even of the arteries. The proportion of glucose in the hepatic vein is said not to have, in any degree, exceeded that in the vessels entering the liver. In this case, this substance would appear to have found its way from the stomach and alimentary canal into the circulation; for in animals fed exclusively on flesh, no glucose could be detected in the vena portarum by the same observer. In experiments performed by Mr. Bernard and Mr. Leconte, and at which the writer assisted, glucose did not appear in the blood of the portal vein, if care were taken to prevent its *reflux from the liver*, whilst it was abundantly furnished by that of the hepatic vein. The animals in these, as in the above case, had been on an exclusive diet of flesh for many months. This would appear conclusive as to the liver originating the glucose. The first experiments of Mr. Poggiale confirm what might have been expected; that in animals living on a mixed alimentation, the food will also furnish sugar in the blood. Hence there would be for man a double source. In herbivora, it is stated by Mr. Colin, of Alfert, that sugar exists in the blood, in the chyle and in the lymph, the saccharine portions of the food furnishing them to the portal veins and chyliferous vessels. He further adds, however, that in the carnivora proper, he found that an animal diet furnished sugar to these vessels during digestion?

The strongest opposition to Mr. Bernard's experiments has recently been made by Mr. Figuier, Assistant Professor in the School of Pharmacy, in Paris, and it is to him that his recent rejoinders have been directed. This gentleman had asserted, as a fact, that the presence of albuminose may interfere with the precipitation of oxide of copper in a fluid containing glucose, (the test of Bernard and Barreswill) and that it is the existence of this principle in the portal blood, which masks the presence of grape sugar. He precipitates the albuminoid substances by alcohol, and recommends the addition of a small portion of acetic acid, to neutralize the carbonate of soda present. With these precautions he insists that glucose can be detected not only in the blood of the portal vein, but in that of the entire system. Mr. Figuier says that he has verified these facts by more than thirty carefully conducted experiments; and he further adds, that for equal weights, the liver can be said to contain scarce twice the amount of glucose to be found in other parts of the body. According to this gentleman, in Bernard's experiments, the flesh of beef and mutton, on which the animals were fed, contains glucose, and thus the substance sought was administered to them in their food.

True, it is, that in Mr. Bernard's doctrine of digestion, albuminose derived from the digestion of meats, is conveyed by the portal vein to the liver, and is there reconverted into Albumen, which does not, like albuminose, mask the reactions of the salt of coppei and potassa with glucose and heat.

The liver, in the opinion of Mr. Figuier, is a reservoir in which animal sugar is stored up, not manufactured; and he strengthens his view by the following experiments:

A dog, which had been fed exclusively on animal food, was killed *two hours* after the last meal; that is, during digestion. The blood entering and leaving the liver was examined; that of the portal vein gave for 100 pts. 0.248 of glucose; that passing from the liver gave but a trace. Repeating the same experiment *four hours* after the last meal, that is, when digestion was more advanced, analysis gave:—

For the blood of the portal vein, (100 pts.) glucose 2.31
" " veins beyond the liver, " 0.304

In an animal kept fasting, the blood of the portal veins gave not a trace, whilst that of the hepatic vein was rich in glucose. In the circle, then, of the circulation, says the observer, the supply obtained from the liver is consumed in respiration, and a fresh amount is furnished to the blood each time that it passes through that organ.

The discrepancy in the results obtained show the want of additional tests for glucose; the fermentation test is useful only where there is a notable proportion of this substance present. The changes of color exhibited by the copper test may be more or less intense, and give rise to uncertain conclusions. The commission appointed to decide on the question have pronounced against Mr. Figuier's analysis, as he did not make use of the fermentation test as well as the others in his experiments.

Longet has also found that the presence of completely digested albuminoid substances masks the reaction of the copper test with glucose. He says that he detected, by the fermentation test, this principle in the blood of the portal vein, in cases where it was not revealed by the double salt of copper and potassa, and even when it had been detected in the alimentary canal and in the hepatic vein by the ordinary means. Hence he considers that some further method of experimenting must be found before the glycogenic function of the liver, based upon the evidences furnished by the present chemical reagents, can be unqualifiedly adopted. His language is: "When the product of the transformation of an azotized aliment by the gastric juice is in considerable quantity, and the glucose in small proportion, the tartrate of copper and potassa, potassa, the polarimeter, alcoholic fermentation, in a word, none of the means now in use, can demonstrate positively the existence of this saccharine principle."

Mr. Lehman has come forward to support Mr. Bernard's view by his own experiments. He invariably makes use of the fermentation test, in addition to the others, and finds that the blood of the portal vein in dogs that have been kept fasting or fed only on meat, does not contain the slightest trace of glucose; a vegetable diet causes its appearance in these vessels in small proportions only. In horses fed on grain the proportion was also very small; but in both horses and dogs, glucose was abundant in the blood of the veins leaving the liver. The other experiments of Mr. Lehman show also conclusively that sugar disappears in the blood, and that the further we proceed from the liver in the circulation, the proportion decreases. It is most abundant in the hepatic vein, and in that of the vena cava, above the junction of that vessel, but in the right side of the heart it becomes intermingled with the blood of the vena cava descendens, which contains no sugar, and thus the proportion is there diminished. It can then be traced into the lungs, where it generally disappears.

The liver, then, is a secretory organ having a double function—that of producing bile and also sugar. The secretion of sugar is a true secretion, not an excretory function, as that of urea by the kidneys; for in these organs urea is found in the blood entering them, but not in that which flows from them, the reverse of that which occurs in the liver with regard to glucose. That this secretion may be considered as under the control of the nervous influence, is well shown by the experiments of Bernard, and since repeated by others, that if the floor of the fourth ventricle be punctured in an animal an artificial diabetes mellitus is established.

This well known fact is most important to be borne in mind, as it may afford a clue to the comprehension of the pathological state of this function. There must be in health an equilibrium between the production and the destruction of glucose. If more is supplied to the lungs than they can get rid of, it will appear in the blood beyond the pulmonary apparatus, and be removed from the general circulation of the kidneys. This is frequently, and even constantly occurring in the normal state. There is, after a mixed meal, the fabrication of glucose to a greater amount than during fasting, and more than is consumed in respiration, and during digestion we may have a temporary glycosuria. Patients should be examined, then, whilst fasting, and the examinations repeated and an average result obtained before we can determine on the amount of disease. The strong sympathetic relation between the glycogenic function of the liver and respiration, is abundantly proved. If the pneumogastric nerve be cut and the extremity nearest the brain be irritated, there takes place an excessive production of glucose, as indicated in the urine; there is an impression made on the cerebral centre, and reflected through the fibres of the sympathetic accompanying the vessels of the liver which influences the production of this principle. The impression made is similar to the excitation arising from the demands of the lungs.

This sympathy, though demonstrable, is not really explained. We see a confirmation of it in diabetes millitus, in which death so generously occurs from pulmonary apoplexy or phthisis. In the triad of the liver, the lungs and the nervous centres, it is difficult to isolate the incipient phenomena, so as to ascertain the starting point of the disease. There is an unfortunate dearth of cadaveric reports on this subject. The disease in question is not a common one; its commencement is often insidious and unheeded, and it is rare that we have the consecutive phenomena described *ab initio*. That lesions of the nervous system may give rise to diabetes mellitus, has been observed by Doctor Goolden, who has collected some very interesting cases—one in particular—in which it followed a blow on the back of the neck. This and the others were relieved by directing remedies to this portion of the system: and a case has fallen under our notice, in which a patient first exhibited symptoms of a spinal adynamia, followed by pulmonary disease and asthma, and in whom the urine subsequently gave evidence of abundant glucose.

The medulla oblongata was in this instance apparently the first at fault in the chain of morbid phenomena. More extended pathological observations may throw light on the order of events in the physiological problem. That the causes assigned by Prout are insufficient, and that the liver can produce glucose from the nitrogenized material conveyed to it by the blood is unquestionable. Lehman's experiments would show that in any event the amount of sugar conveyed in the portal vein under any diet, is but a fractional part of that which leaves the liver, and corroborates Bernard's doctrine.

In the dearth of pathological information, it is gratifying to be able to refer to some facts recently put forth by so reliable an authority as Andral. In eight autopsies of persons who died of diabetes mellitus, he found the liver redder and more full of blood than usual; it was also more uniformly red, showing the increased functional activity of that organ. He adds the following interesting statements as regards the results of dietetic treatment. Entire abstinence from food will diminish, and sometimes suppress, the appearance of sugar in the urine; but he found also that a change of diet, whether it be the addition of vegetable aliment or its entire subtraction, will at first produce a reduction of the amount of sugar, but this will afterwards regain its usual standard.

Lehman having found that the blood leaving the liver, and which abounded in sugar, was deficient in fibrin and hæmatosin, considered that glucose was formed at the expense of these nitrogenized elements. He succeeded in obtaining from hæmatosin a substance which gave reactions similar to glucose, which confirmed him in the opinion. Frierichs had a similar hypothesis. The opinion that fatty matter furnished the material, is no loger sustained. Rollo withheld from his patients all food not of this nature; the sugar was diminished, but the individuals subjected to this regimen were in similar conditions with those who practised perfect abstinence. There can be no doubt but that sugar yielding food will contribute to the glycogenic production, though not to the extent previously imagined. Bernard states that if amylaceous or saccharine nutriment be administered to a dog, the proportion of sugar in the liver will not be increased materially, but there will appear an opalescent emulsive fluid, the result of the metamorphosis of the starchy materials.

It does not necessarily appear, however, that in disease the saccharine matters in the duodenum, the result of digestion, may not pass into the portal vein, be unaffected by the liver, and be thus added to the amount secreted by that organ.

So late as the 24th of September of the present year, Mr. Bernard has brought forward a paper in which he discards all the hypothesis as regards the elements in the blood, &c., from which sugar is produced in the liver. He says that it is the *tissue* of the liver itself which furnishes the materials, and adduces as proof the following experiments:

Having carefully removed the liver of a healthy dog, he fastened a water cock to the trunk of the portal vein, and allowed water to flow with force through the ramifications of that vessel, and thus through the organ itself, escaping by the hepatic veins. The first portion which passed through yielded sugar abundantly; this was continued until the fluid became colorless and ceased to yield sugar; in fact, the liver was completely washed out. The decoction of a portion of the *liver* thus treated gave no sign of sugar. The other portion of the viscus was laid aside for 24 hours, at the end of which time a stream of water was passed through it, which gave abundant evidences of glucose, both by the copper test and fermentation.

This experiment would prove, says the author, that in the liver, in its physiological state, "there are two substances, viz: 1st, sugar, very soluble in water, and which is carried off the blood in washing. 2d. Another substance sufficiently insoluble to remain in the hepatic tissue after this had been deprived of its sugar and of its blood by protracted washing. It is this last substance in the liver, laid aside, which becomes, by degrees, converted into glucose by a species of fermentation."

He has, by further experiments, shown that this matter is insoluble in alcohol and ether. He was unable to detect it in the blood of the portal veins. He considers this discovery as of great value in settling the glycogenic function of the liver, and concludes by the following remarks:

"I wish to show that the sugar formed in the liver is not produced (d'emblee) at once, without intermediate processes in the blood, but that its presence is constantly preceded by a special material deposited in the tissue of the liver, and which immediately precedes its formation." He calls the attention of chemists to the intermediate formations.

Of the use of glucose in the animal economy, there is no absolute demonstration. Its disappearance in the lungs informs us that it is consumed in respiration. Its transformation into lactic acid is readily conceivable, but is simply hypothetical. That the lactates become converted into carbonates, which are again decomposed by the pneumonic acid of the lungs, the $CO^2$ being set free, is the modern doctrine of physiology. Like the other hydrocarbons and fatty matter, its office must be intimately connected with the production of animal heat.

It is a fact worthy of observation, that the livers of birds, (particularly the palmipedes,) which abound in fat, contain also a very large proportion of glucose. In human fatty livers, glucose is always in excess. From the preceding review of the most recent facts and investigations on this subject, we may gather the following conclusions:

That sugar is a normal product in man.

That this principle is secreted in the liver, and that it is a normal function of that organ.

That the source of its supply is from nitrogenized elements.

That the food furnishes it also to the system.

That in the glycogenic function there is a sympathy of relation between the liver, the lungs, and the cerebral centre.

That in the disease called Diabetes Mellitus, the equilibrium of the production and destruction is disturbed, and that any one of these three structures may be at fault; and that it is to one or more of them that our remedies must be directed.

That the experiments of Lehmen, Bernard, and Andral will warrant the careful allowance of small portions of vegetable food in this disease, and thus relieve our patients from one of the most distressing and trying attendants of the present mode of treatment.

That the labors of the physiologists, and above all, of Mr. Claude Bernard, have paved the way for a better understanding of diabetes mellitus, by demonstrating the condition of the glycogenic function in the state of health; but that close and more extended pathological observations are called for to render his researches available to the physician for a successful plan of treatment of a disease which is rare, but has thus far proved intractable.—*Medical Examiner*.

